# Development and Application of Colloidal Gold Test Strips for the Rapid Detection of Canine Brucellosis

**DOI:** 10.3390/bios14080388

**Published:** 2024-08-10

**Authors:** Pengxiang Sun, Xinmei Yang, Jinyue Liu, Yanqing Bao, Jingjing Qi, Xiangan Han, Guanhui Liu, Shaohui Wang, Mingxing Tian

**Affiliations:** 1 Shanghai Veterinary Research Institute, Chinese Academy of Agricultural Sciences, Shanghai 200241, China; 18153537133@163.com (P.S.); 15178100449@163.com (X.Y.); liujinyue0630@163.com (J.L.); ybao@shvri.ac.cn (Y.B.); qjingjing@shvri.ac.cn (J.Q.); hanxgan@shvri.ac.cn (X.H.); 2 College of Life Science and Food Engineering, Hebei University of Engineering, Handan 056038, China; liuguanhui@hebeu.edu.cn

**Keywords:** colloidal gold test strips, canine brucellosis, rapid detection

## Abstract

Brucellosis is a global problem, with the causative agent being the genus *Brucella*. *B. canis* can cause undulant fever in dogs, which is a zoonotic disease that can spread not only among dogs but also to humans. This poses a public health threat to society. In this study, a rapid and straightforward immune colloidal gold test strip was developed for the diagnosis of canine brucellosis through the detection of anti-LPS antibodies in serum samples. Rabbit anti-canine IgG conjugated with colloidal gold was employed as the colloidal gold-labeled antibody. The extracted high-purity R-LPS was employed as the capture antigen in the test line (T-line), while goat anti-rabbit IgG was utilized as the capture antibody in the control line (C-line). The colloidal gold strip exhibited high specificity in the detection of brucellosis, with no cross-reaction observed with the common clinical canine diseases caused by Canine coronavirus (CCV), Canine distemper virus (CDV), and Canine parvovirus (CPV). In comparison to the commercial iELISA kit, the sensitivity and specificity of the colloidal gold test strip were found to be 95.23% and 98.76%, respectively. The diagnostic coincidence rate was 98.47%. The findings of this study indicate that colloidal gold test strips may be employed as a straightforward, expeditious, sensitive, and specific diagnostic instrument for the identification of canine brucellosis, particularly in resource-limited regions.

## 1. Introduction

The number of dogs in our society continues to grow, and pets have become an important part of our lives. Canine brucellosis, which is primarily caused by *Brucella canis*, is a global issue. The bacteria can infect host cells and survive and replicate within them, exhibiting a greater affinity for the cells of the host’s reproductive and immune systems [1]. The clinical presentation of dogs infected with *B. canis* is variable, ranging from asymptomatic cases to miscarriage, orchitis, epididymitis, prostatitis, discitis, fundositis, or lymphadenopathy. The transmission of pathogens among dogs typically occurs through sexual intercourse, aerosols, or direct contact with pathogen-contaminated material on mucous membranes or compromised skin. This has been recognized as a major cause of economic losses in kennels with infected dogs [2,3,4]. In humans, *B. canis* can cause undulant fever, splenomegaly, lymphadenopathy, and, if left untreated, endocarditis and diseases of the central nervous system. Therefore, canine brucellosis is considered a public health threat and requires vigilance [5]. 

*B. canis* is more likely to be detected during acute infections. During the chronic infection period, false positives are the main concern with serology assays due to both nonspecific and specific cross-reactions with shared surface antigens on various bacteria, including *Pseudomonas aeruginosa*, *Bordetella bronchiseptica*, *Actinobacillus equuli*, *Streptococcus*, *Staphylococcus*, and *Moraxella*-type organisms [6]. Various testing methods associated with *B. canis* include traditional isolation and identification [7], polymerase chain reaction (PCR) [8], immunohistochemistry (IHC) [9], recombinase polymerase amplification (RPA) [10], rapid slide agglutination test (RSAT) [11], enzyme-linked immunosorbent assay (ELISA) [12], and the immune colloidal gold technique (GICT) [13]. The most reliable method for detecting *B. canis* is through isolation culture. Unfortunately, *B. canis* has a high affinity for steroid-secreting tissues, making the isolation of *Brucella* from blood extraordinarily difficult in long-standing and chronic cases [14]. The detection rate of PCR in diagnosing *B. canis* is 90%. However, it can only serve as a supplementary diagnostic tool due to its complexity, making it unsuitable for rapid clinical diagnosis [15,16]. There are reports confirming that the sensitivity of the widely used RSAT is only 70% [17]. In addition, no serological test is 100% accurate until 12 weeks after infection, when a strong antibody response persists [18]. GICA is a distinctive immunoassay technique that was developed in the early 1980s. This method is easy to use, does not require specialized personnel, and the results are not affected by temperature or time. It is portable and can be used for diagnosing and screening canine brucellosis at any location. It is particularly suitable for rapidly detecting suspected samples and large numbers of samples in clinical settings [19]. 

Some studies have indicated that the immunodominant antigens of *Brucella* are divided into outer membrane proteins (OMPs) and lipopolysaccharide (LPS). The OMPs most commonly used are Omp28/BP26, Omp25, and Omp31. While these OMPs are conserved in *Brucella*, they are also present in other bacteria. Therefore, using OMPs as antigens will result in significant cross-reactivity [20,21]. This is precisely because the genus *Brucella* evolved from the *Proteobacteria*, which are ubiquitous in nature. However, in comparison to OMPs, LPS is considerably specific as an antigen. It is noteworthy that antibodies directed against the C/Y epitope of the O-antigen have the capacity to cross-react with *Yersinia* species. However, it is advantageous that *Brucella canis* is one of the few *Brucella* species in the genus *Brucella* in which LPS naturally lacks the O-antigen [22]. This suggests that the binding epitope of *B. canis* rough LPS (R-LPS) is solely the core oligosaccharide. Despite the fact that the core epitope exhibits robust specificity for the detection of *B. canis*, the low immune response resulting from the small molecular weight of the core oligosaccharides necessitates the coating of a larger antigen quantity to detect *B. canis* R-LPS antibodies.

In this study, we developed a rapid assay using the framework of colloidal gold technology. High-purity R-LPS was utilized as the detection antigen, specifically for the detection of canine anti-R-LPS antibodies in serum samples. This method exhibits high specificity and accuracy, rendering it a valuable tool for the detection of canine brucellosis.

## 2. Materials and Methods

### 2.1. Bacterial Strains, Cultural Medium, and Reagents

*B. canis* strain RM6/66 (ATCC 23365) was obtained from the National Center for Veterinary Culture Collection (Beijing, China). Phenol, methanol, potassium carbonate, sodium acetate, anhydrous ethanol, acetic acid (99%), polyvinylpyrrolidone K30, sodium hydroxide, ammonia, silver nitrate, anhydrous citric acid, trisodium citrate, and formaldehyde solution (37–40%) were purchased from Sinopharm Chemical Reagent Co., Ltd. (Shanghai, China). Chloroauric acid, BSA, and periodic acid were purchased from Merck Sigma-Aldrich (St. Louis, MO, USA). Trypticase soy broth was purchased from Becton, Dickinson and Company (BD) (Franklin Lakes, NJ, USA). The NC membrane, glass fiber, and absorbing pad were purchased from Shanghai Jieyi Biotechnology Co., Ltd. (Shanghai, China).

### 2.2. Preparation of B. canis LPS

*B. canis* R-LPS is naturally devoid of the O-antigen in comparison to *Brucella* smooth LPS (S-LPS) of other species. Consequently, R-LPS is structurally deficient in comparison to S-LPS. The extraction method is performed as previously described with some modifications [23]. Briefly, 5 g of wet-weight bacterial cells were suspended in hot distilled water (65 °C), an equal volume of 90% phenol solution was added, and the mixture was stirred continuously at 65 °C for 20 min. Subsequently, the mixture was cooled on ice, and centrifugation was performed at 8000× *g* for 30 min at 4 °C. The protein-containing layer and the phenolic layer (lower phase) were then removed. R-LPS was precipitated by the addition of three volumes of a cold methanol reagent, which consisted of 99% methanol and 1% sodium acetate. The sample was then centrifuged at 15,000× *g* for 30 min, the precipitate was collected and resuspended in 15 mL of water containing DNase I (10 μg/mL) and RNase A (10 μg/mL). The solution was incubated at 37 °C for 2 h, and then proteinase K (TIANGEN, Beijing, China) was added to a final concentration of 100 μg/mL, followed by a water bath at 55 °C for 3 h. Proteinase K was inactivated at 70 °C for 15 min and lyophilized in a lyophilizer Coolsafe 55-4 (LaboGene, Copenhagen, Denmark).

### 2.3. Silver Staining and Coomassie Brilliant Blue Stainin0067

R-LPS was separated by SDS-PAGE and silver staining was performed according to the previously described method with some modifications [24]. Initially, the LPS was oxidized in the gel with 0.8% periodic acid in 40% ethanol and 5% acetic acid at room temperature for 20 min. The gel was then washed three times with distilled water for five minutes, after which it was stained for ten minutes with a freshly prepared staining solution (ammoniacal silver nitrate solution). Following two washes, the gel was developed using the developing solution (0.005% citric acid, 0.02% formaldehyde). The gel was washed three times in distilled water and photographed immediately. To assess the protein contamination in the LPS sample, the gel was stained with Coomassie brilliant blue R250 and decolorized with a decolorizing solution. The lanes were examined for any evidence of protein contamination.

### 2.4. Western Blotting

Purified R-LPS was subjected to 12% SDS-PAGE, followed by transfer to a nitrocellulose (NC) membrane (Merck Millipore, Billerica, MA, USA). The membrane was blocked with 10% skimmed milk in phosphate-buffered saline (PBS) containing 0.05% Tween-20 (PBST) at room temperature for one hour. Following three washes with PBST, the membrane was incubated with rabbit anti-*Brucella* polyclonal antibody (prepared in our lab) at room temperature. The membranes were then incubated with 1:10,000 diluted horseradish peroxidase (HRP) goat anti-rabbit IgG (H+L) (Abcam, Cambridge, MA, USA) for a further two hours at room temperature. Development of the HRP signal was performed with LumiQ universal ECL luminescence solution (Share-Bio, Shanghai, China).

### 2.5. Synthesis of Concentrated Colloidal Gold

Colloidal gold was prepared according to the previous report with some modifications [25]. An aqueous solution of 500 mL trisodium citrate [9 mL of 10% (*w/v*) Na_3_C_6_H_5_O_7_] was heated to boiling point, after which 2 mL of 1.0% (*w/v*) chloroauric acid was added. The reaction solution was simultaneously stirred and maintained at 100 °C for a period of five minutes until the color of the solution transitioned from golden to black and eventually to wine red. The mixture was continuously stirred until it reached room temperature. The precipitate was then collected via gradient centrifugation and subsequently concentrated to one-tenth of its original volume. The dimensions of the colloidal gold particles were quantified by transmission electron microscopy at the Wuhan Servicebio Technology Co., Ltd. (Wuhan, China). The obtained colloidal gold solution can be stored at 4 °C for several months and it was used for conjugation with purified antibody by diluting it appropriately.

### 2.6. Optimization of the Colloidal Gold Test Strip

To explore the optimal pH of colloidal gold labeling, 0, 2, 4, 6, 8, 12, 16, 18, 20, 22, 24, 26, 28, and 30 µL of 10 mM K_2_CO_3_ were added to 1 mL of colloidal gold solution, and then 10 µg of rabbit anti-canine IgG (Bersee, Beijing, China) and 100 µL of 10% BSA were added. Additionally, to explore the optimal amount of colloidal gold-labeled antibody, 5, 10, 15, 20, 25, and 30 µg of rabbit anti-canine IgG were, respectively, added to 1 mL of colloidal gold solution after adjusting the optimal pH. All solutions were incubated at room temperature for 20 min. Finally, 100 µL of 10% BSA was added to block the colloidal gold particles, and the spectral absorption curve for OD400–700 was determined using Agilent BioTeck Synergy H1 Multi-Mode Microplate Reader (Santa Clara, CA, USA). Because colloidal gold is an acidic solution and in an acidic environment, antibodies will be rejected by colloidal gold due to their shared polarity. In an alkaline environment, the antibody assumes an opposite charge and is attracted to colloidal gold. The morphology of colloidal gold at 519 nm is determined by the contrast absorption curve, with the absorption wavelength exhibiting a positive correlation with the particle morphology. During the adjustment of pH and antibody amount, the solution will exhibit unstable behavior, resulting in the condensation or enlargement of colloidal gold particles. If the antibody is successfully labeled, the colloidal gold particle volume will increase and the absorption curve will shift to the right. However, the higher the absorption curve, the better the homogeneity of colloidal gold. Therefore, in order to ensure that the majority of antibodies are successfully labeled by colloidal gold particles, a shift to the right and the maximum peak value will be selected as the labeling condition. The optimal labeling pH or the optimal antibody labeling amount of colloidal gold solution was obtained when the maximum shift of the peak appeared. 

A solution of 30 mL of colloidal gold was prepared, with the pH adjusted to the optimal value, and the antibody labeling method was derived from the above proportions. The colloidal gold-labeled rabbit anti-canine IgG solution was then centrifuged at 10,000× *g* for 15 min. The precipitate was collected and resuspended in 3 mL of 10 mM Tris-HCl (pH 8.5), 1% BSA, and 10% sucrose. The colloidal gold-labeled antibody was evenly sprayed onto a 5 cm × 5 cm piece of glass fiber membrane and then dried at 55 °C for 10 min. The NC membrane (CN95) was blocked for one hour at room temperature using the blocking buffer [10 mM Tris-HCl (pH 8.5), 2% BSA, 0.5% PVP-K30, 5% sucrose (*w*/*v*)]. Thereafter, the membrane was dried at 55 °C for 10 min. Subsequently, the R-LPS was serially diluted across a range of 1, 2, 3, and 4 mg/mL and then microsprayed onto an NC membrane (CN95) using the BioDot XYZ3050 dispense system (Irvine, CA, USA) at a position that allowed the formation of the test line (T-line). The goat anti-rabbit IgG antibody was diluted in PBS at a concentration of 1 mg/mL and then microsprayed onto the same NC membrane at a position that allowed the formation of the control line (C-line). The test strips were then dried at 55 °C for 10 min and stored at 4 °C. Furthermore, a weak *B. canis*-positive serum and a *B. canis*-negative serum, as determined by a commercial iELISA kit (PRBTC, Harbin, China), were utilized to identify the optimal coating concentration of R-LPS and the requisite serum dilutions. The optimal concentration was determined based on the color development intensity of the T- and C-lines.

### 2.7. Preparation of the Test Strip Package

**Figure 1:**
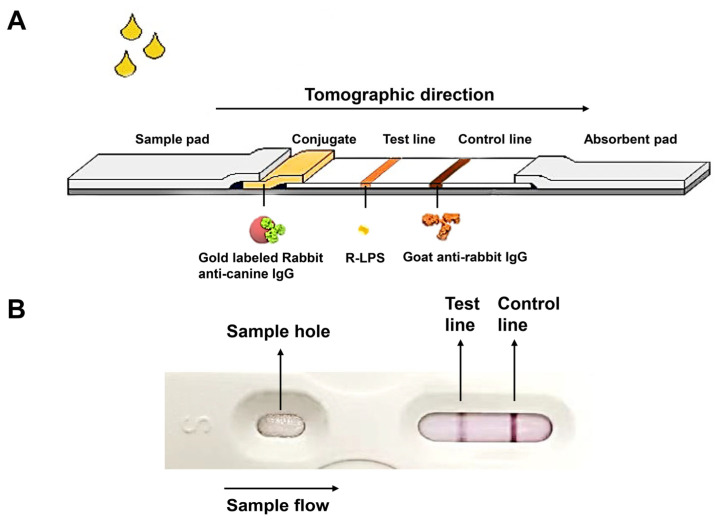
The schematic diagram of the colloidal gold test strip (**A**) and the final product of the colloidal gold test strip package (**B**). The test strip consisted of three pads (sample pad, conjugate pad, and absorbent pad), a nitrocellulose membrane, and a polystyrene backing. The conjugate pads contained gold-labeled rabbit anti-canine IgG. There were two lines on the nitrocellulose membrane: the test line (T-line) and the control line (C-line). The T-line contains R-LPS and the C-line contains goat anti-rabbit IgG antibody.

The immunochromatographic test strip was constructed using the method described by Paek et al. [26]. The colloidal gold-labeled antibody was evenly sprayed on a 5 cm × 5 cm piece of glass fiber membrane and then dried at 55 °C for 10 min. Goat anti-rabbit IgG (1 mg/mL) and R-LPS (4 mg/mL) in water were jet-positioned onto an NC membrane using the BioDot XYZ3050 dispense system as two discrete zones, one designated for the control (C-line) and the other for the test (T-line). Furthermore, an absorbing pad was positioned at the opposite end. Subsequently, the polystyrene backing board was cut into a 4 mm wide and 6 cm long strip (Figure 1). Finally, each strip was placed in a plastic case, each of which was stored individually in desiccated plastic bags. The test results were determined by the number and position of the detection lines on the test strips. The canine antibody introduced to the sample pad will bind with the rabbit anti-canine IgG and be transported in the direction of the absorbent pad by the diluent. In the event that anti-R-LPS antibodies are present, the gold-labeled antibody complex will be retained on the T-line. The remaining gold-labeled antibody complexes will continue to migrate, while the majority of the remaining gold-labeled antibodies will be captured by the goat anti-rabbit IgG at the C-line. Ultimately, the residual antibodies and diluent will be absorbed by the absorbent pad. In the case of positive samples, a single band was observed at the C-line, while a single band was also observed at the T-line. In contrast, negative samples yielded a single band at the C-line, but no band at the T-line. In the event that no band was observed at the C-line, the results were deemed invalid and were therefore discarded.

### 2.8. Specificity and Sensitivity of the Colloidal Gold Test Strip

To assess the specificity and sensitivity of colloidal gold, a series of tests was conducted using sera from dogs with common diseases caused by Canine distemper virus (CDV), Canine parvovirus (CPV), and Canine coronavirus (CCV) infection. Serum samples with an ELISA titer of ≥1:1024 were subjected to the following testing procedure: A single drop (20–30 μL) of serum sample was added, and the mixture was allowed to stand for 20 s. Subsequently, two or three drops (50–100 μL) of 0.01 M PBS containing 0.05% Tween-20 were added. The values were read after a 10-minute period of standing at room temperature, and the test was repeated three times for each sample.

### 2.9. Stability and Repeatability of the Colloidal Gold Test Strip

To assess the stability of the test strips, they were tested with negative and positive serum samples after being stored at room temperature for six months. Three replicates of each negative and positive group were compared with the newly prepared test strip for color matching.

### 2.10. Clinical Evaluation of the Colloidal Gold Test Strip

A total of 263 canine serum samples were collected from various regions, out of which 22 samples were confirmed to be positive. The commercial iELISA kit and colloidal gold test strips were tested in conjunction, and the coincidence rate, accuracy rate, correct index, and sensitivity were compared.

## 3. Results

### 3.1. Identification R-LPS of B. canis

**Figure 2:**
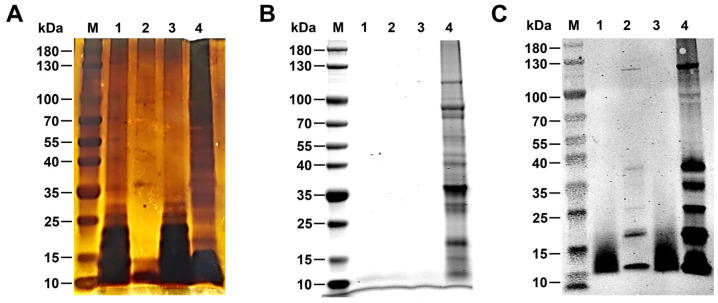
The purity of R-LPS was identified. R-LPS silver staining (**A**); schematic representation of Coomassie Brilliant blue R250 staining (**B**); identification of the R-LPS by western blotting using rabbit anti-*Brucella* antibody (**C**). Lane 1 represents the aqueous phase following hot phenol–water extraction. Lane 2 depicts the phenol phase after proteinase K treatment. Lane 3 is the aqueous phase treated with proteinase K. Lane 4 is the phenol phase after hot phenol–water extraction.

R-LPS was analyzed by silver staining. As illustrated in Figure 2A, bands were observed at approximately 15 kDa in all samples extracted from the phenol phase or aqueous phase. This location corresponds to the position of Lipid A and the core oligosaccharide, confirming the successful extraction of R-LPS. However, R-LPS of *B. canis* was predominantly observed in the aqueous phase (Figure 2A, lanes 1 and 3), with a minor quantity present in the phenolic phase (Figure 2A, lanes 2 and 4). To exclude protein contamination, all extracted LPS samples were separated by SDS-PAGE and stained by Coomassie Brilliant blue R250. The results demonstrated that the R-LPS samples extracted from the aqueous phase, phenol phase by proteinase K digestion, and aqueous phase by proteinase K digestion exhibited no discernible protein contamination (Figure 2B, Lanes 1–3). However, the R-LPS extracted from the phenol phase without proteinase K treatment exhibited notable protein bands (Figure 2B, Lane 4). Western blotting analysis revealed that all R-LPS samples exhibited a clear reaction with anti-Brucella serum (Figure 2C, Lanes 1–4), indicating that the R-LPS has a high reactivity with Brucella-positive serum. However, the R-LPS samples from the phenol phase, with or without proteinase K digestion, displayed protein bands by western blotting analysis (Figure 2C, Lanes 2 and 4). Therefore, the R-LPS extracted from the aqueous phase with proteinase K digestion exhibited an optimal yield and purification, rendering it suitable for subsequent experiments.

### 3.2. Identification of Colloidal Gold Solution

**Figure 3:**
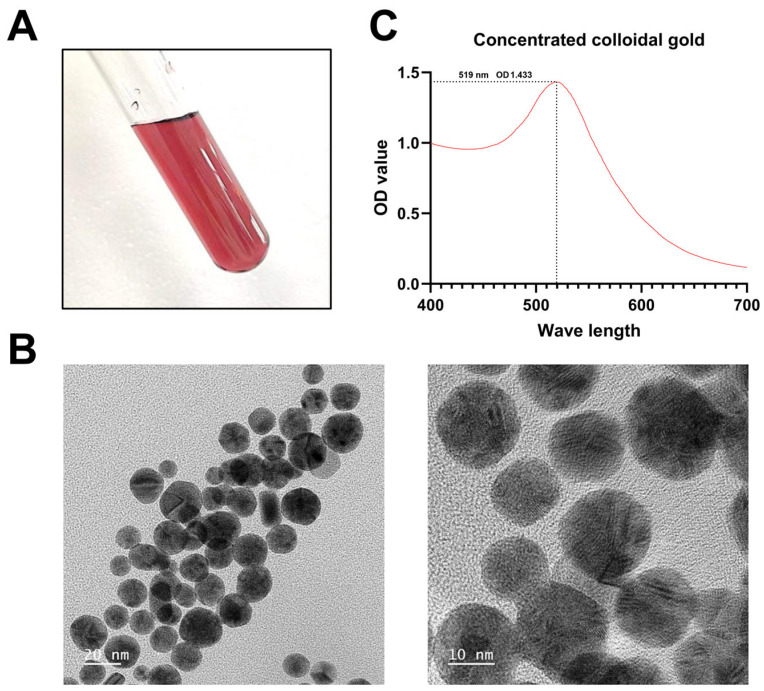
Identification of colloidal gold solution. The colloidal gold solution exhibited a wine-red coloration (**A**). Transmission electron microscopy revealed that the gold particles were predominantly within the range of from 18 to 25 nanometers, exhibiting a relatively uniform morphology (**B**). The peak at 519 nanometers observed in the OD400–700 nm scanning further substantiated that the colloidal gold particles exhibited a relatively small diameter, thereby confirming the high degree of specificity (**C**).

The colloidal gold solution exhibited a wine-red color when observed under an eye view (Figure 3A). Transmission electron microscopy revealed that the majority of the gold particles had a diameter of 18–25 nm, with a relatively uniform morphology (Figure 3B). Furthermore, the peak value was observed at 1.433 at 519 nm, which also demonstrates that the nano-diameter of colloidal gold is small and the specificity is high (Figure 3C). The results indicated that the colloidal gold probe was successfully prepared.

### 3.3. Optimal Labeling Conditions for Colloidal Gold

**Figure 4:**
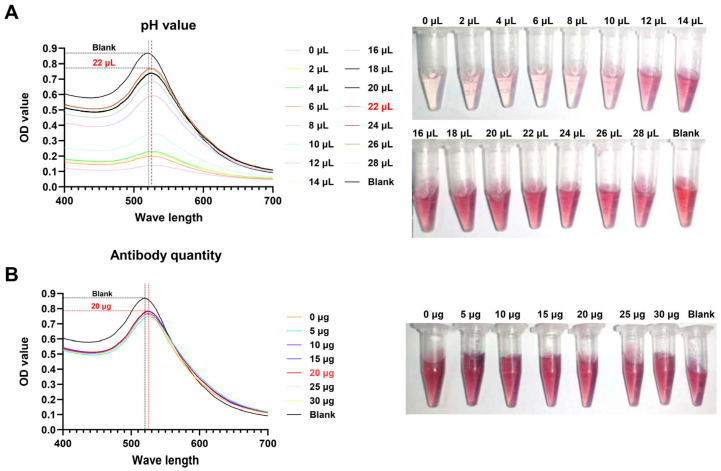
Optimal labeling conditions for colloidal gold. (**A**) The optimal pH for labeling colloidal gold solutions; (**B**) the optimal amount of antibody for labeling colloidal gold solutions.

In order to identify the optimal pH value for labeling a colloidal gold solution, different quantities of K_2_CO₃ solution were added to 1 mL of the same labeled colloidal gold solution. The color change of the colloidal gold solution was observed, and the absorption peak curve was drawn by scanning the solution under OD400–700 nM. The results demonstrated that the color change of the colloidal gold solution was discernible when 2–16 µL of a 0.2 mol/L K_2_CO_3_ solution was added. Concurrently, a notable shift was observed in the absorption peak curve (Figure 4A). However, when 16–28 µL of K_2_CO_3_ solution was added, the color change of the colloidal gold solution was not readily apparent, and the change in the absorption peak curve was minimal (Figure 4A). Although the change in value of the scanning curve is relatively modest, it is evident that the peak of the curve undergoes a significant shift when 22 µL of K_2_CO_3_ is added (Figure 4A). At this juncture, the pH value of the colloidal gold solution was determined to be 8.2 through the use of pH test paper. Subsequently, the optimal amount of antibody labeling in colloidal gold solution at pH 8.2 was further evaluated. As illustrated in Figure 4B, the color of the colloidal gold solution exhibited a slight change with the increased quantity of antibody. Although the color change was not discernible to the unaided eye, a discernible difference in the peak of the OD400–700 nM absorption curve was observed. The greatest shift in the peak of the curve was observed when the quantity of antibody added reached 20 µg (Figure 4B). The data presented above indicate that the optimal pH for labeling colloidal gold solutions is 8.2, and that the optimal amount of antibody labeling is 20 µg/mL.

### 3.4. Optimal Antigen Coating and Serum Dilution for Colloidal Gold Test Strips

**Figure 5:**
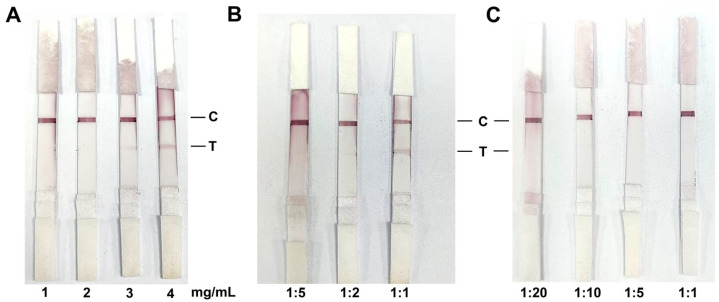
Optimal antigen coating and serum dilution for colloidal gold test strips; (**A**) 1 mg/mL, 2 mg/mL, 3 mg/mL, or 4 mg/mL R-LPS was coated on NC membrane and positive serum was used for detection. (**B**) The positive serum was diluted 1:5, 1:2, and 1:1 to test the optimal dilution concentration. (**C**) The negative serum was tested at dilutions of 1:20, 1:10, 1:5, and 1:1, and none of them was detected as positive.

R-LPS was diluted to concentrations of 1, 2, 3, and 4 mg/mL and coated onto NC membranes at 1 μL/cm using the BioDot XYZ3050 dispense system. The results demonstrated that, when the NC membrane was coated with R-LPS at concentrations of 1 and 2 mg/mL, no discernible band was observed in the T line. Even at a concentration of 3 mg/mL, only a slight band was visible in the T line. However, at a concentration of 4 mg/mL, a significant band was evident in the T line (Figure 5A). However, when the R-LPS concentration exceeded 4 mg/mL, the detection line exhibited a non-reactive false positive band on the NC membrane due to the high coated concentration. Furthermore, the optimal serum dilution for colloidal gold test strips was evaluated. *B. canis*-positive sera were diluted at ratios of 1:1, 1:2, and 1:5 to assess the optimal dilution in test strips. The results demonstrated that a 1:1 dilution ratio was the most efficacious, with the T line clearly discernible to the unaided eye. After dilution to a ratio of 1:2 and 1:5, the T line was difficult to observe (Figure 5B). In addition, a negative serum was also tested in this study, and the test strips demonstrated negative results at any dilution (Figure 5C). These data indicated that the optimal serum dilution ratio is 1:1.

### 3.5. Evaluation of the Specificity of Colloidal Gold Test Strips 

**Figure 6:**
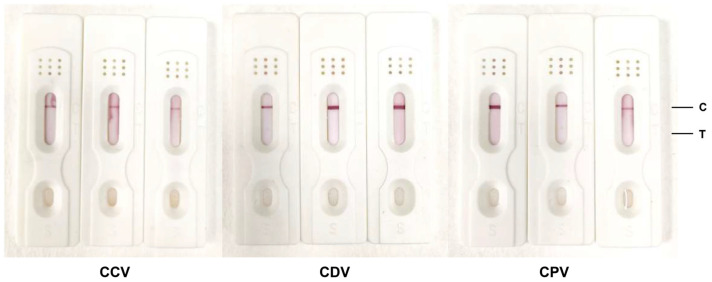
Evaluation of the specificity of colloidal gold test strips. Positive serum samples from clinical dogs infected with Canine coronavirus (CCV), Canine distemper virus (CDV), and Canine parvo virus (CPV) were tested negative in test strips.

To determine the specificity of the test strips, positive serum samples were obtained from clinical canines infected with CCV, CDV, and CPV. The data demonstrated that the test strips established in this study did not exhibit cross-reactivity with CCV-, CDV-, and CPV-positive serum samples (Figure 6), thereby substantiating the test strips’ high specificity.

### 3.6. Comparative Analysis of Colloidal Gold Field Samples with Commercial iELISA

Two hundred and sixty-three clinical dog sera were collected from different regions of China. The commercial iELISA kit (PRBTC, Harbin, China) was used to detect *B. canis* infection in 263 clinical samples, which showed 21 positive cases and 242 negative cases, yielding a positivity rate of 7.98% (21/263). In addition, the colloidal gold test strip established in this study was also used to detect these 263 clinical samples. The result is shown in Table 1, 23 were positive and 240 were negative, with a positive detection rate of 8.75% (23/242). According to the relevant formula shown in Table 2, the relevant parameters of the colloidal gold test strip were calculated. The sensitivity and specificity of the colloidal gold test strip were 95.23% and 98.76%, respectively, and the correct index was 93.99% (Table 2). The coincidence rate between the colloidal gold test strip and iELISA was 98.47%. This result showed that the colloidal gold diagnostic test strip developed in this study has a good clinical detection effect.

## 4. Discussion

In veterinary clinical diagnosis, the timeliness of test results is of paramount importance. Therefore, there is an urgent need for the development of inexpensive, rapid, sensitive, simple, and accurate detection methods. The immune colloidal gold detection method is particularly well-suited to this application, offering test results within 10–15 min and the ability to store test paper for months at room temperature [27]. It has been reported that the five outer membrane proteins BP26, Omp16, Omp2b, Omp25, and Omp31 of *B. canis* are immunogenic and can be used for the detection of *B. canis* infection. In particular, the BP26 protein exhibits a negative value of ELISA, as low as 0.3, while the negative value of the fusion protein synthesized as described in the aforementioned report reaches as high as 0.48 [20]. This value is highly dependent on the microplate reader detection, making it challenging to distinguish positive and negative serum samples with the naked eye. However, chronic long-term infection will result in a very low antibody titer in serum, which is susceptible to appearing near the critical value. This can lead to a significant number of misdiagnosed cases, causing significant challenges to clinical screening [20]. Consequently, the outer membrane protein was not selected as the coating antigen in this study; instead, R-LPS was selected. One advantage of R-LPS is the lack of O-antigen, which results in fewer R-LPS epitopes and less cross-reaction. This makes it a more promising antigen for diagnosis. However, due to the lack of O-antigen, the molecular weight of R-LPS was significantly reduced, which resulted in a weaker immunogenicity than that of S-LPS and a weaker antigen–antibody binding reaction [28]. In order to achieve satisfactory detection results, it was necessary to coat a greater number of antigens in the actual detection process, a finding that was also verified in this study. In comparison to the 0.5–2 mg/mL of common antigen-coated concentration, the coating amount of R-LPS reached 4 mg/mL, which also demonstrated its weak antigen reactivity. 

In this study, although the colloidal gold strip demonstrated a high degree of concordance in the detection of 263 samples, there are still instances where sera with low antibody concentrations may be overlooked. It is notable that, among the negative samples, three false-positive sera were identified by the test strips, suggesting that R-LPS may have cross-reacted with certain pathogens, which is consistent with the results previously reported. Consequently, in dogs exhibiting clinical signs of reproductive system problems, the initial screening using colloidal gold test strips yields positive results, which should be confirmed through laboratory retesting. Due to the limited number of clinical samples, this study has only demonstrated that this test strip does not produce cross-reactivity with common clinical viral diseases infected by CCV, CDV, and CPV. Further studies are necessary to confirm that the canine *Brucella* test strips do not produce false positive results for other bacterial diseases, such as *Bordetella bronchiseptica*, *Actinobacillus equuli*, and *Moraxella*-type organisms in dogs.

In a study conducted by Keid et al. [29], a commercial ICT test was employed for the diagnosis of canine brucellosis. The findings revealed that the diagnostic sensitivity of the ICT kit in a group of *B. canis*-infected dogs was 89.58%, and the diagnostic specificity of this ICT in a group of *B. canis*-non-infected dogs was 100%. However, 10.41% of the positive dogs were not identified by this ICT kit, resulting in a high misdiagnosis rate. One potential explanation for this result is the use of different coated antigens. In the commercial ICT kit, the R-LPS is co-extracted with outer membrane proteins as coated antigens. This is due to the fact that R-LPS is embedded between outer membrane proteins, which results in the epitopes of core oligosaccharides being blocked by the outer membrane protein [30]. This results in the binding epitopes being derived primarily from the outer membrane proteins. In this study, R-LPS was extracted and coated as the sole antigen. The specificity of the colloidal gold test strip was 98.76%, and the sensitivity was increased to 95.23% in tested serum samples, achieving an optimal balance, which was more suitable for primary screening of a large number of clinical samples.

## 5. Conclusions

In this study, a colloidal gold strip was successfully established for the detection of canine brucellosis infected by *B. canis*. The strip is inexpensive, rapid, sensitive, simple, and accurate. In comparison to the commercial iELISA kit, the present method demonstrated a sensitivity of 95.23%, a specificity of 98.76%, and a diagnostic compliance rate of 98.47%. The test can be utilized for the preliminary clinical screening of *B. canis* infection, thereby offering a convenient instrument for the prevention and control of canine brucellosis.

## Figures and Tables

**Table 1:** Detection of colloidal gold test strips.

Colloidal Gold Detection	Positive	Negative	Total
Test Positive	20 (a)	3 (b)	23 (a + b)
Test Negative	1 (c)	239 (d)	240 (c + d)
Total	21 (a + c)	242 (b + d)	263 (a + b + c + d)

**Table 2:** Formula for evaluating colloidal gold properties.

Evaluation Indicators	Formula for Assessment	Results
Positive	a + c	21
Negative	b + d	242
False Positive	b	3
False Negative	c	1
Sensitivity (A)	a/(a + c) × 100%	95.23%
Specificity (B)	d/(b + d) × 100%	98.76%
Misdiagnosis	b/(b + d) × 100%	1.23%
Underdiagnosis	c/(a + c) × 100%	4.70%
Diagnostic Compliance rate	(a + d)/(a + b + c + d) × 100%	98.47%
Correctness Index (Youden)	A + B − 1	93.99

Note: “a–d” represents the number of samples in Table 1 that tested positive or negative for colloidal gold strips, respectively.

## Data Availability

Data are contained within the article.
